# New Insights into the Pathogenesis of Metabolic-Associated Fatty Liver Disease (MAFLD): Gut–Liver–Heart Crosstalk

**DOI:** 10.3390/nu15183970

**Published:** 2023-09-14

**Authors:** Keungmo Yang, Myeongjun Song

**Affiliations:** Division of Gastroenterology and Hepatology, Department of Internal Medicine, College of Medicine, The Catholic University of Korea, Seoul 06591, Republic of Korea; yang27jin@catholic.ac.kr

**Keywords:** metabolic-associated fatty liver disease, gut microbiota, cardiovascular disease, gut–liver–heart crosstalk

## Abstract

Metabolism-associated fatty liver disease (MAFLD) is a multifaceted disease that involves complex interactions between various organs, including the gut and heart. It is defined by hepatic lipid accumulation and is related to metabolic dysfunction, obesity, and diabetes. Understanding the intricate interplay of the gut–liver–heart crosstalk is crucial for unraveling the complexities of MAFLD and developing effective treatment and prevention strategies. The gut–liver crosstalk participates in the regulation of the metabolic and inflammatory processes through host–microbiome interactions. Gut microbiota have been associated with the development and progression of MAFLD, and its dysbiosis contributes to insulin resistance, inflammation, and oxidative stress. Metabolites derived from the gut microbiota enter the systemic circulation and influence both the liver and heart, resulting in the gut–liver–heart axis playing an important role in MAFLD. Furthermore, growing evidence suggests that insulin resistance, endothelial dysfunction, and systemic inflammation in MAFLD may contribute to an increased risk of cardiovascular disease (CVD). Additionally, the dysregulation of lipid metabolism in MAFLD may also lead to cardiac dysfunction and heart failure. Overall, the crosstalk between the liver and heart involves a complex interplay of molecular pathways that contribute to the development of CVD in patients with MAFLD. This review emphasizes the current understanding of the gut–liver–heart crosstalk as a foundation for optimizing patient outcomes with MAFLD.

## 1. Introduction

Metabolic dysfunction-associated fatty liver disease (MAFLD) has become a major worldwide health issue, prompting a shift in the perception and classification of nonalcoholic fatty liver disease (NAFLD) [1]. While NAFLD primarily focuses on the accumulation of liver fat, MAFLD considers both liver fat and associated metabolic risk factors, such as diabetes, dyslipidemia, or obesity, thus providing a more detailed approach to diagnosing and managing fatty liver disease. Therefore, the introduction of the term MAFLD reflects a more comprehensive approach to capturing the diverse spectrum of patients affected by this condition and acknowledges the intricate interplay between metabolic abnormalities and liver health [1,2]. MAFLD poses a substantial economic burden, as well as implications for clinical diagnosis, management, and the molecular mechanisms that link it to end-stage liver disease or cancer [3,4]. Its pathogenesis is complex and involves various factors, such as hepatic immune cells, insulin resistance, gut-related factors, and lifestyle choices [5]. Further research is needed to gain a deeper insight into the pathogenesis, prognosis, and therapeutic options for MAFLD.

Multiorgan crosstalk plays a vital role in the development and advancement of various liver diseases, including NAFLD and alcohol-associated liver disease (ALD) [6,7]. Various organs, including the heart, kidneys, intestines, lungs, and pancreas, interact with the liver through complex signaling pathways and molecular mediators [8]. This crosstalk is mediated by factors such as hepatokines, myokines, extracellular vesicles, and immune cells, with dysfunctional crosstalk leading to multiorgan diseases and complications [7,8]. Adiponectin and fibroblast growth factor 21 have been investigated as important mediators of crosstalk between the liver and adipose tissue [9,10]. Additionally, immune cells, such as T cells, B cells, natural killer (NK) cells, and hepatic macrophages, contribute to interorgan crosstalk in liver diseases [11,12]. Remarkably, the gut–liver axis has also been implicated in various liver disease progression including MAFLD [13,14,15]. Moreover, the liver can influence the pathophysiology of other organs, such as the kidneys or heart, and vice versa [6,8]. Understanding the intricate interplay between different organs and their contributions to liver disease is essential for developing effective therapeutic strategies.

The interaction between the gut and the liver is pivotal in the development and evolution of MAFLD. Dysbiosis of the gut microbiota, alterations in intestinal barrier function, and metabolic endotoxemia are some factors that play an important role in the onset of MAFLD [16,17]. The gut microbiota and their metabolites, such as bile acids and short-chain fatty acids (SCFAs), have been identified in the regulation of liver lipid metabolism and inflammation in MAFLD [17,18]. The gut–liver axis is involved in the modulation of hepatic mitochondrial function, glycolipid metabolism, and oxidative stress in MAFLD [19,20]. It is affected by factors such as diet, proton pump inhibitor use, and obesity, which can influence the progression of MAFLD [21,22]. Therefore, understanding the complex interplay between the gut and the liver is essential for elucidating the mechanisms underlying MAFLD and developing targeted therapeutic strategies.

MAFLD is closely associated with cardiovascular disease (CVD), a condition that involves disorders of the blood vessels and heart, such as stroke, heart attack, and other related issues. The incidence of CVD is higher among patients with MAFLD, and it is considered a potent risk factor for the development of CVD [23,24]. The association between MAFLD and CVD is related to shared risk factors, including diabetes, obesity, metabolic syndrome, and hepatic inflammation [24,25]. Additionally, MAFLD can lead to hepatic fibrosis, which can worsen congestion and cardiac function, potentially leading to heart failure [24]. The gut–liver axis, endothelial dysfunction, oxidative stress, hepatic insulin resistance, altered lipid metabolism, and systemic inflammation are among the pathophysiological mechanisms linking MAFLD and CVD [26]. Overall, the association between the liver and heart diseases in the context of MAFLD highlights the need for a comprehensive cardiovascular risk assessment and the development of targeted therapeutic approaches [27].

This review aims to provide novel insights into the pathogenesis of MAFLD, explore the gut–liver–heart crosstalk in MAFLD, and outline the therapeutic approaches for managing MAFLD. It will also emphasize comprehending the mechanisms involved in the onset and progression of MAFLD, as well as the interplay among the gut, liver, and heart in this disease.

## 2. Pathophysiology and Molecular Mechanism of MAFLD

**Figure 1:**
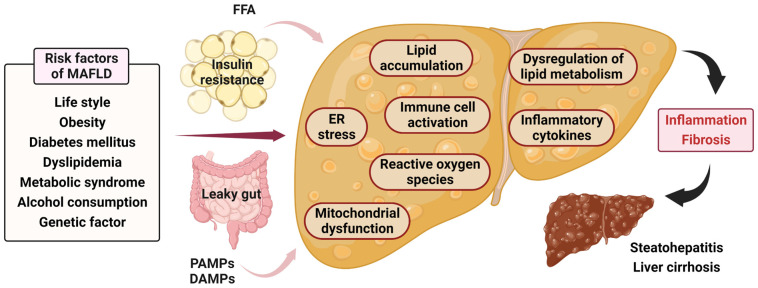
Pathophysiology of metabolic dysfunction-associated fatty liver disease (MAFLD). FFA, Free fatty acid; PAMP, Pathogen-associated molecular pattern; DAMP, Damage-associated molecular pattern; ER, Endoplasmic reticulum.

MAFLD is a complex condition with multifactorial pathophysiology involving various molecular mechanisms and immune system dysregulation. The pathogenesis of MAFLD are affected by genetic, environmental, and lifestyle factors [28]. Its pathophysiology is mainly associated with abnormalities in oxidative stress, lipid metabolism, insulin resistance, inflammation, gut microbiota dysbiosis, and immune system activation [29,30] (Figure 1).

One major factor contributing to the onset of MAFLD is systemic insulin resistance [31]. Insulin resistance leads to activated lipolysis in the adipose tissue, resulting in the release of free fatty acids (FFAs) into the bloodstream. These FFAs are absorbed in the liver and undergo de novo lipogenesis, resulting in the accumulation of triglycerides (TGs) in hepatocytes, which is a hallmark of MAFLD [4,6,32]. Excessive accumulation of TGs in the hepatocytes is known as hepatic steatosis and is the initial stage of MAFLD [32]. Steatohepatitis is specified by hepatocyte ballooning, inflammation, and hepatic fibrosis. The exact mechanisms underlying the transition from simple steatosis to steatohepatitis are not comprehensively understood but are believed to involve multiple factors, such as oxidative stress, mitochondrial dysfunction, and dysregulated lipid metabolism [33].

Oxidative stress also plays a crucial role in the pathogenesis of MAFLD [32]. Excessive accumulation of FFAs in the hepatocytes leads to the production of reactive oxygen species, which can cause oxidative damage to hepatocytes and promote inflammation [31]. Mitochondrial dysfunction has also been associated with the pathophysiology of fatty liver. Impaired mitochondrial function and increased mitochondrial fission have been observed in MAFLD, leading to increased oxidative stress and hepatocyte injury [34]. Endoplasmic reticulum (ER) stress is another important mechanism involved in the development of MAFLD [33,35]. ER stress arises when the ER’s capacity to handle protein folding is exceeded, leading to the activation of the unfolded protein response. Chronic ER stress can contribute to hepatocyte injury, inflammation, and fibrosis in MAFLD [33,35].

Inflammation has an essential role in the progression of MAFLD from steatosis to steatohepatitis [31]. Activation of pattern recognition receptors (PRRs) by pathogen- and danger-associated molecular patterns (PAMPs and DAMPs) leads to the activation of immune cell and inflammatory change in MAFLD [12,29,31]. Inflammatory cytokines, including interleukin-6 (IL-6) and tumor necrosis factor-alpha (TNF-α), are upregulated in MAFLD and contribute to hepatocyte injury and fibrosis [12,36]. Dysregulation of lipid metabolism is another key feature of MAFLD. Abnormalities in lipid uptake, synthesis, oxidation, and export contribute to lipid accumulation in hepatocytes and the progression of hepatic steatosis [37]. Dysregulation of lipid metabolism can also lead to the production of toxic lipid metabolites, including diacylglycerols and ceramides, which can promote inflammation and hepatocyte injury [37].

## 3. Gut–Liver Axis in MAFLD

### 3.1. Gut Dysbiosis in MAFLD

Gut dysbiosis, characterized by a disbalance in the gut microbiota composition, has been implicated in the progression of obesity and MAFLD [15,32,36,38]. Several studies have investigated the molecular mechanisms underlying gut dysbiosis in patients with metabolic disorders. In obesity, gut dysbiosis is related to changes in the diversity and composition of the gut microbiota. Dysbiosis can lead to increased energy harvesting from diet, metabolic disturbances, and inflammation associated with obesity [39], as dysbiosis in obesity is defined by a decrease in beneficial bacteria and an increase in harmful bacteria [40]. Similar to that in patients with obesity, gut dysbiosis has been associated with the pathogenesis of MAFLD. Dysbiosis in MAFLD is characterized by a decrease in beneficial bacteria, such as Akkermansia and Prevotella, and an increase in potentially pathogenic bacteria [41,42]. This dysbiosis can contribute to increased gut permeability, leading to the translocation of bacterial products, such as lipopolysaccharides (LPSs), into the liver, triggering inflammation and liver injury [41]. Furthermore, dysbiosis is able to have an effect on bile acid metabolism, leading to alterations in lipid metabolism and the development of hepatic steatosis [42].

Dysregulation of gut microbiota in obesity and MAFLD is influenced by various factors, such as life style, diet, and host genetics. Dietary patterns, including high-fat diets or diets rich in processed foods, can promote dysbiosis and contribute to the development of obesity and MAFLD [43]. Lifestyle factors, like physical inactivity and stress, can also impact the gut microbiota composition [44,45]. Additionally, host genetics can influence the susceptibility to gut dysbiosis and its associated metabolic disorders [46,47]. Understanding the molecular mechanisms underlying gut dysbiosis in these metabolic disorders will provide insights into potential therapeutic strategies for their management. However, additional research is required to demonstrate the specific mechanisms and interactions involved in gut dysbiosis and to develop targeted interventions for gut dysbiosis in obesity and MAFLD.

Gut dysbiosis also has an essential role in the development of ALD. Chronic alcohol consumption is accompanied by changing gut microbiota composition, leading to dysbiosis [13,48]. Dysbiosis in ALD is specified by reduced bacterial diversity, changes in the abundance of specific bacterial taxa, and an increase in the number of potentially pathogenic bacteria [13,49]. It is associated with increased gut permeability, leading to the translocation of bacterial products into the liver [50]. This triggers an inflammatory response and immune reaction, which contributes to alcohol-induced liver injury and disease progression. Dysbiosis can also affect bile acid metabolism, leading to changes in lipid metabolism and the development of hepatic steatosis [36]. Chronic alcohol consumption can directly affect the gut microbiota composition by enhancing the growth of alcohol-tolerant bacteria and reducing the growth of beneficial bacteria [51]. Additionally, alcohol metabolism can generate toxic metabolites, such as acetaldehyde, which can further contribute to gut dysbiosis [52]. Dysbiosis can also affect the gut–liver axis, influencing hepatic inflammation, fibrosis, and ALD progression [36]. Additionally, it can affect the immune system, alter immune responses, and promote liver injury [13].

### 3.2. Mechanism of Gut–Liver Axis in MAFLD

#### 3.2.1. Intestinal Permeability

**Figure 2:**
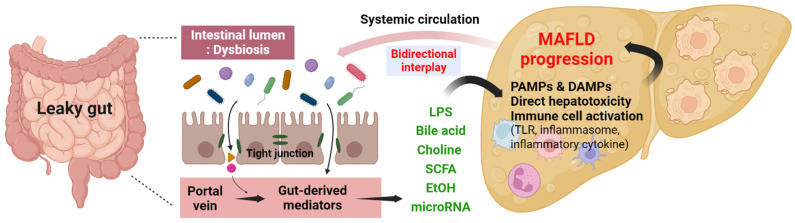
Pathogenesis of gut–liver axis. LPS, Lipopolysaccharide; SCFA, Short-chain fatty acid; EtOH, Ethanol; PAMP, Pathogen-associated molecular pattern; DAMP, Damage-associated molecular pattern; TLR, Toll-like receptor; MAFLD, Metabolic dysfunction-associated fatty liver disease.

Intestinal permeability, usually referred to as “leaky gut”, is a phenomenon characterized by an increased passage of substances from the gut lumen into the bloodstream in MAFLD (Figure 2) [15]. It has also been implicated in the pathogeneses of both NAFLD and ALD [13,15]. Dysregulation of intestinal permeability in these liver diseases is related to alterations in the gut microbiota composition, disruption of tight junctions in intestinal epithelium, and increased prevalence of small intestinal bacterial overgrowth (SIBO) [11,50]. In MAFLD, increased intestinal permeability has been observed, allowing the translocation of bacterial products into the liver. This triggers an inflammation and immune response, contributing to liver injury and disease progression [53]. Several previous studies have shown that patients with MAFLD exhibit compromised intestinal tight junction integrity and disorganized microvilli in the gut mucosa [41,54]. The disruption of the gut barrier and subsequent endotoxemia contribute to the progression and development of MAFLD [55].

Similarly, in ALD, increased intestinal permeability has a critical role in the pathogenesis of the disease. According to a previous study, significant damage to the intestinal epithelium occurs only when ethanol is administered at a very high dose in a single instance [56]. Chronic alcohol consumption can directly affect the gut microbiota composition and disrupt the intestinal barrier function, leading to increased intestinal permeability and the translocation of bacterial products, including LPS, into the liver [50,57]. Interestingly, upon chronic alcohol consumption, the alteration of the intestinal barrier is attributed to a decrease in the expression of proteins responsible for maintaining tight junctions between enterocytes, including the zonula occludens protein ZO-1 and occludin [58,59]. Alcohol-induced intestinal hyperpermeability and endotoxemia contribute to liver inflammation and injury [60,61]. Dysbiosis of the intestinal microbiota and SIBO has also been identified in ALD, further contributing to increased intestinal permeability [62].

#### 3.2.2. Metabolites Produced by the Gut Microbiome

Bile acids, synthesized in the liver and additionally metabolized by the intestinal microbiota, have a crucial role in the gut–liver axis [15,36]. The gut microbiota contribute to bile acid metabolism by converting primary bile acids into secondary bile acids [63]. Microbial modification of bile acids is essential for maintaining a healthy microbiota, insulin sensitivity, lipid and carbohydrate metabolism, and innate immunity [64]. Bile acids also act as signaling molecules by binding to nuclear receptors, like farnesoid X receptor (FXR) and G protein-coupled bile acid receptor (TGR5), in the gut and liver [64,65]. These receptors play crucial roles in regulating bile acid synthesis, transport, and metabolism. Activation of FXR and TGR5 by bile acids influences glucose and lipid metabolism, inflammation, and immune responses [66,67,68]. Experimental studies have shown that alterations in bile acid signaling can affect liver diseases, including MAFLD and hepatocellular carcinoma (HCC) [69]. The gut–liver axis is bidirectional, with bile acids influencing the intestinal microbiota, which, in turn, modulates bile acid composition and metabolism [70]. This interplay among bile acids and gut microbiota is crucial for maintaining gut homeostasis and overall health.

Endogenous ethanol, produced in the gut through microbial fermentation and liver metabolism, is involved in various aspects of gut–liver communication and liver pathophysiology [15]. Endogenous ethanol is immediately removed from portal blood by alcohol dehydrogenases (ADHs) and other enzymes [71]. However, when ADHs are inhibited, blood alcohol levels can increase [71]. Preclinical and human studies have investigated increased levels of endogenous ethanol, acetaldehyde, and acetate in liver diseases, such as MAFLD [72]. Endogenous ethanol can lead to increased portal endotoxemia, impaired gut barrier function, and upregulated signaling pathways, all of which contribute to the pathophysiology of MAFLD [73]. Additionally, endogenous ethanol induces mitochondrial dysfunction and contributes to liver damage [73]. Ethanol production appears to be influenced by the abundance of Proteobacteria, particularly Escherichia coli or Klebsiella pneumoniae, in the gut microbiome and the availability of carbohydrates from the diet [74]. Interestingly, the administration of Klebsiella pneumoniae through oral gavage or fecal microbiome transplantation into normal mice induces MAFLD [74].

Choline, an essential nutrient, has a substantial effect on the gut–liver axis through its metabolism and interaction with the gut microbiota [15]. Choline is altered by the intestinal microbiota into trimethylamine (TMA), which is subsequently converted into trimethylamine N-oxide (TMAO) in the liver [75]. Studies have shown that TMAO exacerbates atherosclerosis and is closely correlated with its severity in humans [76]. The composition of the microbiota influences TMA production from choline, and low levels of colonization by TMA-producing bacteria can reduce choline availability [75]. Additionally, the microbiota can modulate the bioavailability of dietary choline, thereby affecting its role in very low-density lipoprotein (VLDL) synthesis, insulin resistance, glucose homeostasis, hepatic lipid export, and liver health [53]. Choline metabolism by the gut microbiota has also been linked to the regulation of bile acid metabolism, energy utilization, inflammation, and fat deposition [77].

SCFAs are metabolites produced by the intestinal microbiota by the fermentation of dietary fiber. These metabolites have been implicated in various physiological processes of MAFLD [11,15] and are related to the regulation of hepatic lipid metabolism, including the inhibition of lipogenesis and the promotion of fatty acid oxidation [78]. SCFAs can also act as signaling factors by interacting with G-protein-coupled receptors (GPCRs), like GPR43 or GPR41, expressed on various cell types in MAFLD [79]. Activation of these receptors by SCFAs can modulate immune responses, regulate gut barrier integrity, and influence lipid metabolism [36]. Bacteria-producing SCFAs, including Megasphaera, Bifidobacterium, Prevotella, and Butyrivibrio, are more abundant in individuals with MAFLD than in healthy individuals [15]. This leads to increased levels of SCFAs in the feces of MAFLD patients [80]. Furthermore, patients with advanced fibrosis have been observed to have elevated levels of fecal acetate, whereas those with mild or moderate hepatic fibrosis have increased amounts of propionate and butyrate [79,81].

### 3.3. Gut-Derived Signaling Pathway in MAFLD

The gut–liver axis is a complicated system that involves communication and interaction between the gut and liver (Figure 2). It has an essential role in diverse physiological processes, including immune responses and inflammation in MAFLD [8,12]. The signaling pathways of the gut–liver axis are mediated by the recognition of PRRs, DAMPs, and PAMPs [36,82]. Additionally, alterations in the composition and diversity of the intestinal microbiota can affect the production of metabolites, which can influence MAFLD progression [83]. The NLRP3 inflammasome is a multimeric protein complex that plays a pivotal role in the inflammatory response to MAFLD and is activated by diverse stimuli, such as DAMPs or PAMPs [84]. Activation of the NLRP3 inflammasome leads to the modulations of proinflammatory cytokines, such as IL-18 or IL-1β. The release of these cytokines contributes to the initiation of the inflammatory reaction [84,85].

Inflammatory signaling pathways in the gut–liver axis involve the activation of various immune cell types. Innate immune cells including Kupffer cells in the liver and lymphoid cells (ILCs) in the gut play important roles in recognizing and responding to PAMPs and DAMPs [13,86]. For example, ILCs in the gut produce IL-22, which mitigates alcohol-induced hepatic injury and prevents gut permeability caused by alcohol [57,86]. Toll-like receptors (TLRs) are important components in the immune system that play a critical role in the development of MAFLD. They detect PAMPs and DAMPs that are released during cellular damage or infection [87]. TLR4 is well known for its role in recognizing LPS, one of the critical factors from Gram-negative bacteria, and in triggering inflammatory responses [87]. In MAFLD, TLR4 signaling has been involved in the initiation of local inflammatory change and the development of hepatic steatosis [88,89]. TLR4 deficiency or down-regulation in hepatocytes has been identified to resolve hepatic inflammation and improve insulin resistance and steatosis [88]. In contrast, the activation of TLR9 induces the expression of proinflammatory and antiviral cytokines through the adaptor protein MyD88 [90]. In a previous report, mice lacking TLR9 were observed to exhibit improved steatohepatitis and liver fibrosis compared to normal mice, which was attributed to the inhibition of the MyD88 pathway [91].

MAFLD is influenced by various immune cells, including T cells, dendritic cells, and NK cells, through the gut–liver axis [14,38,92]. The accumulation of specific T-cell populations in the liver is associated with different stages of MAFLD and can have proinflammatory effects [93]. Homeostasis of the gut microbiota directly influences the stimulation of intrahepatic T-cell subsets [93]. Additionally, regulatory T cells have been found to increase in frequency in MAFLD-related HCC, indicating the role of intestinal microbiota in regulating immunity in an HCC microenvironment [94]. Changes in the microbiota can also affect the translocation of antigenic components, significantly influencing the immune system in the liver. For instance, an altered gut microbiota, as observed in a hepatic fibrosis, can result in the reconfiguration of the T-cell receptor immune repertoire, reducing its diversity and limiting the range of antigens that these cells can recognize [95]. Additionally, the equilibrium between endotoxins and exotoxins that enter the liver can influence the immune response, particularly by affecting certain subsets of T cells with innate-like characteristics [93].

## 4. Liver–Heart Axis in MAFLD

### 4.1. Relationship between MAFLD and Heart Diseases

**Figure 3:**
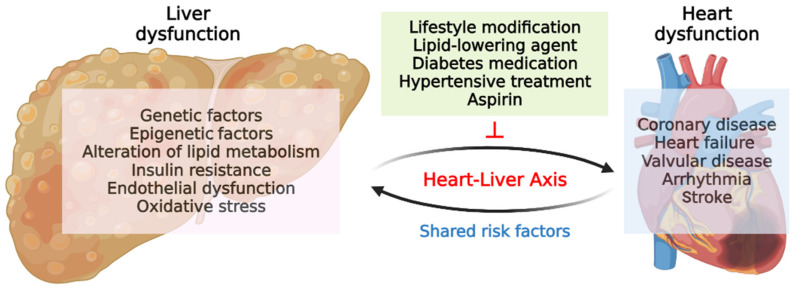
Pathogenesis, cardiac manifestations, and therapeutic approaches in liver–heart axis.

The association between MAFLD and CVD is well established owing to the sharing of numerous risk factors in their pathophysiology [96] (Figure 3). Compared to patients without MAFLD, those with MAFLD appear to have a significant risk of cardiovascular events, including myocardial infarction, angina, or stroke [97]. Epidemiological evidence supports the link between MAFLD and subclinical atherosclerosis as well as an elevated prevalence of CVD [96]. A previous report has also shown that MAFLD is related to increased carotid artery thickness [98,99], arterial wall stiffness [100,101], and impaired endothelial vasodilation [102]. In type 2 diabetes patients, MAFLD is independently related to a higher prevalence of coronary, cerebrovascular, and peripheral vascular disease [23,96]. Among patients who underwent coronary angiography, the presence of MAFLD was associated with the severity of CAD [103,104]. A previous meta-analysis and systematic review of a large number of participants further support the association between MAFLD and atherosclerosis, hypertension, and CVD [105].

Advanced fibrosis in MAFLD worsens the severity of CAD, and the MAFLD stage is related to the development of coronary atherosclerosis, particularly in the case of mixed-type plaques. As expected, severe hepatic steatosis increased the risk of CAD and mixed atherosclerotic plaques [106]. MAFLD is also linked to an increased risk of developing acute coronary syndromes [107]. Noncalcified plaques were associated with MAFLD, whereas the calcified plaques did not significantly differ between individuals with and without MAFLD [108]. Noncalcified plaques indicate a higher susceptibility to acute coronary events, suggesting a potential mechanism for sudden cardiac events in patients with MAFLD. These findings emphasize the importance of considering MAFLD as a significant risk factor for cardiovascular complications and highlight the need for effective management strategies to mitigate cardiovascular complications associated with MAFLD.

MAFLD is also associated with congestive heart failure, which increases the risk of incident heart failure [109]. The presence of epicardial fat in MAFLD patients results in irregular energy metabolism in the left ventricle, which contributes to diastolic dysfunction [110]. MAFLD is also an critical risk factor for congestive heart failure with reduced ejection fraction, even after considering obesity and insulin resistance [111]. The combination of MAFLD and heart failure poses a greater risk of higher mortality [112].

Valvular heart disease (VHD) is a known complication of MAFLD [113]. Several previous reports have shown a strong relationship between MAFLD and valvular heart diseases, such as mitral annulus calcification or aortic-valve sclerosis [113,114,115,116]. The exact mechanisms underlying the association between MAFLD and valvular heart disease are not comprehensively understood. One hypothesis suggests that hypertension, increased arterial stiffness, and hyperuricemia may contribute to the progression of valvular heart disease in patients with MAFLD [117]. Another hypothesis suggests that the release of proinflammatory and profibrogenic factors from the liver in MAFLD may have a role in the pathophysiology of cardiac complications, such as heart failure or valvular heart disease [118].

Atrial fibrillation (AF) is a common cardiac arrhythmia in MAFLD patients, particularly in those with type 2 diabetes [119,120]. One study followed a random sample of 400 type 2 diabetes patients over 10 years and found that MAFLD was related to a significantly increased risk of AF [119]. This relationship remained significant after adjusting for sex, age, hypertension, and electrocardiographic features [119]. Another study conducted a meta-analysis utilizing observational studies and found that MAFLD was associated with an increased risk of AF [121]. Shared risk factors and pathological mechanisms, such as obesity and proinflammatory or oxidative states, may contribute to the progression of AF in patients with MAFLD [122]. Similarly, other life-threatening arrhythmias have also been found to occur more frequently in patients with MAFLD. In a large cohort study, an excessively prolonged QT interval was observed according to the severity of MAFLD [123]. Another retrospective study showed a significantly increased risk of persistent heart block in MAFLD patients as compared to those without MAFLD [120]. In brief, MAFLD can present with cardiac manifestations ranging from subtle alterations in the cardiac structure and function to more significant cardiovascular complications. Therefore, early detection and comprehensive management of both liver disease and cardiovascular risk factors are crucial for improving the outcomes in individuals with MAFLD. Figure 3 briefly summarizes the liver–heart axis and cardiac manifestations.

### 4.2. Gut Microbiota and Liver–Heart Axis in MAFLD

As mentioned previously, the alteration of intestinal microbiota is significantly related to the pathogenesis of MALFD development. Despite microbiota being a critical component involved in the pathophysiology of MAFLD, the most crucial factor determining the clinical outcome of MAFLD is cardiac manifestations, such as CVD. According to recent studies, MAFLD is an important risk factor for CVD, with a substantially increasing prevalence of CVD [25,109] (Figure 3). Interestingly, dysregulation of the intestinal bacterial microbiota in patients with CVD and MAFLD could be a crucial contributing factor influencing the severity of metabolic dysfunction [124]. In patients with CVD and MAFLD, there was a notable increase in the abundance of *Copococcus* and *Veillonella*, whereas the abundance of *Parabacteroides*, *Ruminococcus*, *Bacteroides,* and *Bifidobacterium* decreased [124,125]. In another metabolomic study using 16S rRNA sequencing and serum metabolome profiling, the presence of MAFLD in patients with CVD may lead to poorer clinical outcomes, potentially due to its influence on the characteristics of the microbiota and circulating metabolites [126]. The alteration of the fungal microbiota in patients with MAFLD and CVD may have an important impact on the regulation of these metabolic disorders [127]. Based on these pieces of evidence, it can be inferred that the gut–liver–heart axis may have a pivotal role in the multiorgan crosstalk mechanisms involved in the pathophysiology of MAFLD.

### 4.3. Possible Mechanisms in Liver–Heart Axis

#### 4.3.1. Genetic and Epigenetic Manifestations

Several studies have explored the genetic and epigenetic factors linking hypertension and MAFLD [128]. Genetic polymorphisms in the adiponectin gene (ADIPOQ), which plays a role in glucose and lipid metabolism, have been identified as a potential link between hypertension and MAFLD [129]. Another gene, angiotensin receptor type 1 (AGTR1), has also been associated with MAFLD and liver fibrosis. Epigenetic changes further contribute to the susceptibility of MAFLD, hypertension, and cardiovascular disease by interacting with inherited risk factors [130]. Additionally, genetic forms, such as sterol regulatory element-binding proteins (SREBPs), transmembrane 6 superfamily member 2 (TM6SF2), and patatin-like phospholipase domain-containing protein-3 (PNPLA3), have been found to have a protective effect against CVD in relation to the MAFLD stages [131,132]. The relationship between PNPLA3 and CVD appears to be affected by triglyceride metabolism and MAFLD severity [133,134]. Several gene polymorphisms have been identified to be involved in the MAFLD–CVD relationship, including adiponectin, apolipoprotein C3, leptin receptor, TNF-α, manganese superoxide dismutase, and angiotensin [132,135]. MicroRNA expression analysis revealed altered levels of miR132 and miR-143 levels in patients with both MAFLD and CVD, suggesting their potential use as biomarkers for disease identification and monitoring [136].

#### 4.3.2. Inflammation and Cytokines

Inflammatory responses induced by various cytokines affect the progression of both MAFLD and CVD [137]. MAFLD-related inflammatory changes affect the structure of the coronary wall, leading to coronary artery disease (CAD) and increased CVD mortality [138]. Markers, including C-reactive protein, lipoprotein A, plasminogen activator inhibitor 1, fetuin-A, and homocysteine, are elevated in MAFLD patients and are associated with a high risk of CVD [106,132,139]. Reactive oxygen radicals in MAFLD can induce the production of proinflammatory cytokines, including IL-8, IL-6, IL-1β, or TNF-α, further contributing to the atherogenic stimuli and the inflammatory status in metabolic syndrome [140,141,142]. Additional mechanisms contributing to systemic inflammation include the increased release of VLDLs from overloaded triglyceride-laden hepatocytes, which stimulate TLRs and elevated levels of hepatokines, such as retinol-binding protein 4 and fetuin-A [143,144].

#### 4.3.3. Endothelial Dysfunction

Endothelial dysfunction refers to the compromised function of the endothelium and inner layers of blood vessels, which plays a crucial role in maintaining vascular homeostasis. Patients with MAFLD and CVD exhibit endothelial dysfunction, characterized by impaired vasodilation and increased levels of circulating biomarkers of endothelial activation and dysfunction [102,145]. Endothelial dysfunction is related to increased oxidative stress, as evidenced by increased oxidative stress markers and reduced antioxidant capacity in these patients [145]. MAFLD also exacerbates endothelial dysfunction, leading to a higher risk of cardiovascular complications. Insulin resistance, a hallmark of MAFLD, contributes to endothelial dysfunction by various mechanisms, such as a dysregulation in nitric oxide production [146]. Oxidative stress, another characteristic feature of MAFLD, affects vascular endothelial function and further increases the risk of CVD [132,146].

#### 4.3.4. Lipid Metabolism

Lipid metabolism is also important in the development of MAFLD and CVD [147]. The dysregulation of lipid metabolism, characterized by abnormal levels of circulating lipids, is commonly observed in obesity and metabolic diseases, such as MAFLD and atherosclerosis [148]. In patients with MAFLD and CVD, the accumulation of lipids in hepatocytes (steatosis) progresses to a more advanced stage of inflammation (steatohepatitis), which can lead to hepatic fibrosis and cirrhosis [149]. Lipid storage organelles and lipid-modifying pathways may also play essential roles in nonapoptotic cell death, which is associated with MAFLD [150]. Patients with MAFLD may exhibit reduced levels of VLDLs due to decreased synthesis, which increases the risk of atherosclerosis [151]. Elevated levels of triglyceride-rich lipoproteins are associated with calcified and noncalcified coronary lesions in patients with MAFLD [117,152]. These findings suggest that MAFLD contributes to the progression of CVD by dysregulating lipid metabolism and other metabolic risk factors.

#### 4.3.5. Insulin Resistance

Insulin resistance is a pivotal factor in MAFLD progression in patients with CVD [132]. One study found that insulin resistance, as measured by the homeostasis model assessment, was negatively related to endothelial function in patients with MAFLD and CVD [102]. Insulin resistance in MAFLD is also associated with other metabolic parameters and liver histology. One study demonstrated that the severity of liver histopathology in MAFLD was associated with carotid atherosclerosis, insulin resistance, and metabolic syndrome [98]. Jun N-terminal kinases (JNKs), which are factors of the mitogen-activated protein kinase superfamily, contribute to insulin resistance, particularly to the secretory function and survival of pancreatic β-cell [153]. Activation of JNKs by inflammatory cytokines, like TNF-*α*, can inhibit insulin signaling in hepatic insulin resistance [154]. Additionally, high levels of leptin and glucose can induce the secretion of IL-1β from pancreatic islets, promoting β-cell malfunction and death, which is also mediated by the JNK pathway [154].

#### 4.3.6. Clonal Hematopoiesis in CVD and MAFLD

Clonal hematopoiesis, characterized by somatic mutations in hematopoietic stem cells, has emerged as a fascinating and clinically relevant phenomenon [155]. While initially identified in the context of hematologic malignancies, recent research has illuminated its far-reaching impact on various organ systems, including the liver and cardiovascular system. Clonal hematopoiesis-associated mutations, often involving genes, such as TET2, DNMT3A, and ASXL1, drive a proinflammatory state, leading to chronic systemic inflammation [155,156,157]. This persistent inflammation contributes to endothelial dysfunction, promoting atherosclerosis and increasing the risk of cardiovascular events [157]. Additionally, the liver is also affected by clonal hematopoiesis-related inflammation, potentially exacerbating conditions such as steatosis and liver fibrosis [158]. Therefore, clonal hematopoiesis’s broader impact suggests new possibilities for investigating its connection to inflammation and organ-specific diseases, potentially leading to therapeutic opportunities in the liver–heart axis.

## 5. Therapeutic Approaches in MAFLD

### 5.1. Therapeutic Approaches for Targeting Gut–Liver Axis

Therapeutic approaches for targeting the gut–liver axis in MAFLD involve various strategies aimed at modulating the intestinal microbiota, improving intestinal barrier function, and reducing hepatic inflammation. One strategy involves the use of probiotics, live microorganisms that provide health advantages when ingested in sufficient quantities. Probiotics have been demonstrated to improve hepatic function and reduce inflammation in patients with MAFLD [159,160,161]. They can modulate the microbiota composition, enhance intestinal barrier function, and reduce the translocation of harmful bacteria and their metabolites into the liver [159,162]. On the other hand, prebiotics are indigestible components of food that specifically encourage the proliferation of advantageous bacteria within the gastrointestinal tract. By promoting the expansion of beneficial bacteria, prebiotics can help restore the gut microbial balance to improve liver health [163,164].

Fecal microbiota transplantation (FMT) is another therapeutic strategy that involves the transfer of fecal material from a healthy donor into the gastrointestinal tract of a recipient. FMT diminishes inflammation in the colon and initiates the restoration of intestinal homeostasis by activating immune-mediated pathways [165]. This leads to the production of IL-10 from adaptive and innate immune cells, which ultimately controls intestinal inflammation [165]. A recent clinical trial showed that FMT has the potential to enhance the therapeutic benefits for patients with MAFLD, and its effectiveness in a clinical setting is more pronounced in lean patients with MAFLD as compared to those who had obesity [166]. On the other hand, allogeneic FMT did not result in a decrease in insulin resistance or the proportion of fat in the liver, as measured by magnetic resonance imaging [167].

### 5.2. Therapeutic Approaches for Targeting Liver–Heart Axis

Lifestyle modifications are essential for the treatment of MAFLD and associated risk factors of CVD. Dietary changes and increased physical activity have been shown to have significant effects on multiple risk factors, thereby reducing the risk of CVD [137]. Dietary interventions can target specific components of the Western diet that contribute to the development of CVD and MAFLD. For example, reducing the consumption of dietary L-carnitine and phosphatidylcholine, which are found in large amounts in red meat and other animal products, may help reduce the generation of metabolites associated with CVD events [96]. Physical activity and exercise have been shown to present beneficial effects on both MAFLD and CVD. Exercise improves insulin sensitivity and glucose metabolism, which are important factors in the development and progression of MAFLD. Physical activity has been associated with a decreased risk of all-cause mortality among individuals with MAFLD [168]. Regular exercise can lead to weight loss, which is known to attenuate MAFLD and associated cardiovascular risk factors [168].

Statins are medications commonly prescribed to lower LDL cholesterol levels and diminish the risk of CVD. Previous reports have investigated the potential benefits of statins in patients with MAFLD [113,137] and have been shown to reduce CVD events and mortality in MAFLD [169]. Atorvastatin treatment also reduces CVD morbidity and mortality to a greater extent in patients with abnormal liver enzyme levels caused by MAFLD than in those without MAFLD [170]. Therefore, practical guidelines recommend the use of statins in patients with MAFLD who have dyslipidemia and an increased risk of CVD. The decision to initiate statin treatment should be based on a comprehensive assessment of the cardiovascular risk and the potential benefits of the treatment [171,172].

Pioglitazone has been studied for its potential benefits in MAFLD related to heart disease and has been shown to attenuate histology in patients with MAFLD, including an improvement in lobular inflammation, steatosis, and hepatocyte ballooning [173]. Interestingly, pioglitazone has demonstrated potential benefits in reducing cardiovascular events in patients with type 2 diabetes and has been related to improved cardiac function and altered myocardial substrate metabolism [174,175]. Insulin-sensitizing agents, including metformin, have also been used to treat MAFLD, and metformin improves the clinical outcomes in patients with diabetes and steatosis, hepatic inflammation, bridging fibrosis, or compensated cirrhosis [176]. Further research is required to determine the effects of metformin on CVD outcomes in patients with MAFLD.

The use of glucagon-like peptide-1 (GLP1) receptor agonists has also shown promise in the management of MAFLD and its related cardiovascular complications [137,177]. GLP1 analogs, including liraglutide, have been found to improve body weight and glycemic control in patients with MAFLD. Additionally, GLP1 receptor agonists have been shown to decrease cardiovascular risk in patients with type 2 diabetes, decrease hepatic steatosis, and improve inflammation in patients with noncirrhotic MAFLD [178]. Current evidence suggests that GLP1 receptor agonists are a promising therapeutic option for the treatment of MAFLD and its associated cardiovascular risks. Sodium-glucose cotransporter 2 (SGLT2) inhibitors can also improve steatosis and reduce hepatic triglyceride content in patients with MAFLD and have demonstrated cardiovascular benefits in patients with heart failure and type 2 diabetes [179,180]. Remarkably, according to the recent meta-analysis of clinical trials, GLP1 agonists and SGLT2 inhibitors significantly reduced cardiovascular outcomes in patients with type 2 diabetes [181,182,183]. The above findings imply that the reversal of MAFLD might be an optimal therapeutic option for MAFLD-related cardiovascular complications.

The management of hypertension in patients with MAFLD and CVD is important to reduce cardiovascular complications and mortality [184]. Aggressive management of hypertension, along with other comorbid conditions, like dyslipidemia and hyperglycemia, is recommended to decrease the risk of CVD in these patients [177,185]. Interestingly, angiotensin receptor blockers (ARBs) significantly mitigate lipid metabolism in patients with MAFLD [186]. Similarly, aspirin has been associated with a reduced risk of fibrosis progression in patients with MAFLD [187]. Aspirin modulates bioactive lipids, stimulates the biosynthesis of proresolving mediators, and inhibits proinflammatory lipids, which may help prevent progressive liver damage [187]. The use of aspirin in patients with MAFLD may help mitigate the increased cardiovascular risk [177]. However, the decision to use aspirin in patients with MAFLD and CVD should be individualized, taking into account the risk of bleeding in the patient and the potential benefits of aspirin therapy. In summary, by improving glycemic control, lipid profiles, and blood pressure, these medications can contribute to the overall improvement of MAFLD outcomes and reduce the risk of CVD complications.

According to the diagnostic criteria for MAFLD, the categories can be broadly divided into three: MAFLD, MAFLD cirrhosis, and concomitant MAFLD with other liver diseases [188]. Particularly in the case of concomitant MAFLD with other liver diseases, there are studies suggesting that the outcomes of MAFLD improve when other factors associated with MAFLD, such as alcohol consumption or viral hepatitis, are well managed [188]. For example, there have been reports of significant improvement in hepatic steatosis when antiviral therapy is administered to patients with chronic hepatitis B [189]. Interestingly, in a recent preclinical study, the administration of tenofovir alafenamide in MAFLD animal models showed anti-inflammatory effects by inhibiting the AKT protein activity of intrahepatic immune cells, resulting in serum and histological improvements [190]. These findings could represent a new therapeutic approach that can improve the outcomes of MAFLD and cardiovascular complications in patients with concomitant MAFLD and other liver diseases.

## 6. Conclusions

Multiorgan crosstalk has a crucial role in the pathogenesis of MAFLD. Understanding the intricate interplay among the gut, liver, and heart is important for developing effective strategies for managing MAFLD and its associated complications. Multiorgan crosstalk emphasizes the role of various factors, such as hepatic inflammation, alterations in lipid metabolism, mitochondrial dysfunction, and dysbiosis of the intestinal microbiota, in the development and progression of MAFLD. Among these factors, host–microbiome interactions may have a pivotal role in regulating metabolic and inflammatory processes as potential therapeutic targets in the gut–liver–heart axis. Furthermore, it highlights the association between MAFLD and CVD, emphasizing shared risk factors, endothelial dysfunction, systemic inflammation, dyslipidemia, and insulin resistance as the underlying mechanisms. Therefore, an integrated understanding of these complex interactions provides valuable insights into optimizing patient outcomes through comprehensive approaches for the prevention and treatment of MAFLD.

## Data Availability

Not applicable.
